# Glucose testing and insufficient follow-up of abnormal results: a cohort study

**DOI:** 10.1186/1472-6963-6-87

**Published:** 2006-07-12

**Authors:** Lisa M Kern, Mark A Callahan, David J Brillon, Maryelena Vargas, Alvin I Mushlin

**Affiliations:** 1 Department of Public Health, Weill Medical College, Cornell University, New York, NY, USA; 2 Department of Medicine, Weill Medical College, Cornell University, New York, NY, USA; 3 New York-Presbyterian Hospital, New York, NY, USA; 4 Columbia University School of Nursing, New York, NY, USA

## Abstract

**Background:**

More than 6 million Americans have undiagnosed diabetes. Several national organizations endorse screening for diabetes by physicians, but actual practice is poorly understood. Our objectives were to measure the rate, the predictors and the results of glucose testing in primary care, including rates of follow-up for abnormal values.

**Methods:**

We conducted a retrospective cohort study of 301 randomly selected patients with no known diabetes who received care at a large academic general internal medicine practice in New York City. Using medical records, we collected patients' baseline characteristics in 1999 and followed patients through the end of 2002 for all glucose tests ordered. We used multivariate logistic regression to measure associations between diabetes risk factors and the odds of glucose testing.

**Results:**

Three-fourths of patients (78%) had at least 1 glucose test ordered. Patient age (≥45 vs. <45 years), non-white ethnicity, family history of diabetes and having more primary care visits were each independently associated with having at least 1 glucose test ordered (p < 0.05), whereas hypertension and hyperlipidemia were not. Fewer than half of abnormal glucose values were followed up by the patients' physicians.

**Conclusion:**

Although screening for diabetes appears to be common and informed by diabetes risk factors, abnormal values are frequently not followed up. Interventions are needed to trigger identification and further evaluation of abnormal glucose tests.

## Background

Of the 20.8 million Americans with diabetes, 6.2 million (30%) are unaware of their diagnosis [1]. It is unclear why nearly one-third of patients with diabetes are undiagnosed. One potential reason could be insufficient screening for diabetes in the context of medical care. Insufficient screening may occur, because there is no consensus from national guidelines on who should be screened [2-4]. Patients could also be undiagnosed if screening is done but abnormal values are not recognized and addressed.

The extent of diabetes screening in primary care and the patient characteristics associated with screening are not well understood. Some previous studies were limited by reliance on patients' self-reports of being screened [5,6], which may not be accurate. Other studies considered actual testing, but measured this in populations that were predominantly young [7], had few non-whites [8], or were restricted to managed care settings [8].

Our primary objectives were: 1) to measure the rate of actual glucose testing among patients without known diabetes, using data from a diverse patient population in a mixed payer setting; and 2) to assess the short-term outcomes of testing, including the rate of follow-up for abnormal results. Our secondary objectives were to determine which patient characteristics are associated with any glucose testing and to determine whether the practice patterns observed resemble national guidelines.

## Methods

### Study design and patients

We conducted a retrospective cohort study of patients who were randomly selected from a large outpatient general internal medicine practice affiliated with an academic health center in New York City. This practice has an electronic medical record, which captures all appointments, test orders, laboratory results and progress notes. There was no systematic screening program for diabetes in place at the time of this study, nor did the electronic medical record have specific flags for episodes of diabetes screening. The Institutional Review Board of the Weill Medical College of Cornell University approved this research.

We generated electronically a list of patients who had an initial visit in 1999 with a full-time attending physician (i.e. one who spent ≥80% effort on patient care per week). The rationale for restricting our sample to those with initial, or first, visits was that initial visits often have more complete data for diabetes risk factors, including family history. The alternative approach of tracing existing patients back to their initial visits was considered less feasible (due to transitions from paper to electronic medical records) and more prone to errors in data collection. We randomly sampled from the list of initial visits and reviewed medical records until we found at least 300 patients who met our inclusion criteria: we required that patients have at least 2 additional visits by the end of 2002, and we excluded patients who were younger than 20 years of age, had known diabetes at the initial visit, or were pregnant at any time during the study period. Our target sample size, based on a prior study of self-reported diabetes screening [5], provided 80% power to determine a 15% absolute difference between the rate of glucose testing among patients with 0–1 risk factors and the rate of glucose testing among patients with 2 or more risk factors [9].

### Data collection

Two trained abstractors collected data manually from patients' electronic medical records. Data collected from the initial visit included demographic data, medical history, and physical exam findings. Each patient was then followed over time for all glucose tests ordered in the outpatient setting, until a new diagnosis of diabetes was made or until December 31, 2002, whichever came first. Thus, most patients were followed for at least 3 years, a strategy which is consistent with the screening interval recommended by the American Diabetes Association (ADA) [2] and which was chosen to maximize the study's ability to capture glucose testing for patients. If a primary care physician documented that a patient had been diagnosed with diabetes by an outside physician or during a hospitalization since the last visit, we counted that as a diagnosis and did not require the primary care physician to do further diagnostic testing. We recorded the number of visits and the number of different primary care physicians seen by each patient over the observation period. For each glucose test ordered, we recorded: the type of glucose test; the patients' fasting state during the test; polyuria or polydipsia reported at the time of the test; the physician's stated reason, if any, for ordering the test; the glucose value; the physician's interpretation of the glucose value; and the physician's subsequent action.

### Inter-rater reliability

Using duplicate data collection for a random 10% sub-sample of the patients, we calculated percent agreement between data abstractors. Percent agreement was high for glucose orders (94%), type of glucose test (98%), fasting state (98%), presence of polyuria or polydipsia (98%), and action following glucose testing (100%). Percent agreement was lower for the reason for testing (66%) and for glucose interpretation (74%).

### Statistical analysis

We used descriptive statistics based on complete data to characterize the patients in our sample, the patterns of glucose testing and rates of follow-up. We considered different cutoffs for clinically significant glucose values, based on the ADA's interpretation of fasting values (≥126 mg/dl for diabetes [2], 110–125 mg/dl for the old definition of impaired fasting glucose [10], and 101–125 mg/dl for the new definition of impaired fasting glucose [2,11]). If glucose values were random or were not documented as fasting, we still considered values in these ranges to be potentially abnormal, because they should trigger repeat testing to confirm that a patient's fasting values are in the normal range. When calculating rates of follow-up for abnormal values, we conducted a sensitivity analysis to assess the effect of continuity, restricting the sample to only those who had all visits with the same physician.

We considered 10 potential patient predictors of glucose testing: age, gender, ethnicity, insurance type, family history of diabetes, hypertension, high cholesterol, body mass index (BMI), the number of visits during the study period, and continuity (whether all visits were with the same physician). Only 1 patient had a documented history of gestational diabetes and no patients had a documented history of impaired fasting glucose; thus, these variables were not considered in prediction models. For each variable included in the model, values were missing for <10% of patients, except for ethnicity (missing for 17%) and height (missing for 59%). To address this, we used multiple imputation to generate 5 complete versions of our dataset, with some variation in the imputed values across versions [12,13]. We then analyzed each imputed dataset and combined estimates of odds ratios (OR) and 95% confidence intervals (CI) by applying Rubin's formula [14].

We used bivariate and multivariate logistic regression to measure associations between patient characteristics and the odds of having at least 1 glucose test ordered over the study period. Because the proportion of data missing for height was large, we generated 2 different multivariate models: the first model included weight alone (without height) and the second model included BMI (reflecting both height and weight). Results were similar for the bivariate models and for the first multivariate model whether we used complete or imputed data. We therefore display results based on imputed data.

In addition, we generated a composite variable for the number of diabetes risk factors each patient had [age 45 years or older, non-white ethnicity, family history of diabetes, hypertension, high cholesterol, and overweight or obese (BMI ≥ 25)]. We calculated the odds of glucose testing for each additional risk factor, and we separately calculated the odds of glucose testing for 2 or more risk factors vs. 0–1 risk factors.

We determined the proportions of patients who would be eligible for screening based on the criteria set by national guidelines. Patients were considered eligible for screening by the ADA if they were 45 years of age or older, or were overweight or obese and had 1 or more of the other diabetes risk factors listed above [2]. Patients were considered eligible for screening by the Centers for Disease Control and Prevention (CDC) if they were 25 years of age or older [3], and by the U.S. Preventive Services Task Force (USPSTF) if they had hypertension and/or hyperlipidemia [4,15]. Among those eligible for screening by each guideline, we calculated the proportion that had at least 1 glucose test ordered.

We considered p-values ≤ 0.05 to be significant. Data analysis was performed using Stata 8 (College Station, TX).

## Results

### Patient characteristics

Our electronic search identified 3543 patients as having had initial visits in 1999 with full-time attending physicians. We reviewed medical records for 621 (18%) of these patients, in order to find 301 who met criteria for inclusion. The other 320 (52%) were excluded for the following reasons: did not actually have an initial visit in 1999 (n = 27), did not have 2 additional visits before the end of 2002 (n = 250), age under 20 years (n = 7), known diabetes at the initial visit (n = 19), pregnancy (n = 10) and missing medical records (n = 7).

The 301 included patients together had 19 different primary care physicians. The average time from the first to last visit in the study period was 2.3 years, with an average of 6 primary care visits per patient. More than half of patients (57%) saw their own primary care physician for every visit. Patients had an average age of 40 years (Table 1). Sixty percent were female, and 41% were of minority ethnicity. Most patients (87%) had commercial insurance. Approximately 1 in 5 had a family history of diabetes. Very few women (1%) had a history of gestational diabetes. A history of hypertension (10%) or high cholesterol (8%) was not uncommon. The average height and weight were 67 inches and 162 pounds, respectively, which is equivalent to an average BMI of 25.

### Characteristics of glucose tests

A total of 559 glucose tests were ordered over the study period. The number of tests per patient ranged from 0 to 10, with a mean of 1.9 tests (median 2 tests) per patient. More than three-fourths of patients (78%) had a least 1 glucose test ordered. Of the glucose tests ordered, 90% were part of chemistry panels. For 3% of tests, physicians documented that patients were fasting, and for 11% of tests physicians documented that they were ordered for screening purposes.

Of the 559 glucose tests ordered, 466 (83%) were done. The proportion of potentially abnormal values depended on the cutoff chosen: 65 (14%) were ≥101 mg/dl, 26 (6%) were ≥110 mg/dl, and 15 (3%) were ≥126 mg/dl. Of the 65 potentially abnormal values (≥101 mg/dl), only 1 was obtained following a visit for polyuria or polydipsia.

When the glucose value was 101–109 mg/dl in asymptomatic patients, physicians reported that the value was normal in 35% of cases and did not document any interpretation for the remainder of cases. No documented follow-up occurred for any of these values. When the glucose value was 110–125 mg/dl in asymptomatic patients, physicians recorded that these values were normal (14%) or potentially erroneous (43%), or they did not record any interpretation (43%). For these values, physicians repeated the test (43%) or did not indicate any further action (57%). For the small number of glucose values ≥126 mg/dl in asymptomatic patients, physicians interpreted these as normal (17%), representing diabetes (25%) or potentially erroneous (25%), or they did not document any interpretation (33%). For these values, physicians repeated the test (31%), prescribed a change in diet (15%), prescribed a change in both diet and exercise (8%), or did not indicate any subsequent action (46%). Patients were more likely to have some follow-up action for a glucose value ≥110 mg/dl if every visit was with the patient's primary care physician (69% follow-up rate) than if at least 1 visit was with a covering physician (14% follow-up rate; p = 0.02).

### Predictors of glucose testing and adherence to guidelines

In bivariate models, 7 patient characteristics were statistically significant predictors of glucose testing (Table 2). Four of these remained statistically significant in both multivariate models (Table 2). Older age (≥45 years), non-white ethnicity, family history of diabetes, and having more visits each independently increased the adjusted odds of having at least 1 glucose test ordered. The magnitude of these associations was sizable; for example, being 45 years of age or older increased the odds of glucose testing 12-fold, being non-white increased the odds of glucose testing nearly 4-fold, and having a family history of diabetes increased the odds of glucose testing 3-fold.

Model 1 (adjusting for weight) and Model 2 (adjusting for BMI) were similar, except that female gender reached statistical significance as a predictor of glucose testing only in Model 2, decreasing the adjusted odds of testing (Table 2). Also in Model 2, as BMI increased, the odds of glucose testing increased, but this association did not reach statistical significance. Of note, neither hypertension nor high cholesterol was an independent predictor of glucose testing in either multivariate model.

For each additional diabetes risk factor a patient had, the odds of glucose testing increased 3-fold, adjusting for gender, insurance, number of visits and continuity (OR 3.06, 95% CI 1.99 – 4.70). Patients with 2 or more risk factors were approximately 10 times as likely to have glucose tests ordered as patients with 0 or 1 risk factor, adjusting for gender, insurance, number of visits and continuity (OR 9.57, 95% CI 3.93 – 23.31).

Among patients eligible for diabetes screening according to each national guideline, the rates of glucose testing were high: 96% for the ADA, 82% for the CDC, and 90% for the USPSTF.

## Discussion

In this study of patients without known diabetes, the majority (78%) had 1 or more glucose tests ordered over a 3-year period. One in seven glucose values (14%) was potentially abnormal. When the glucose value was 110 mg/dl or greater, physicians interpreted these as normal 16% of the time and did not indicate plans for any subsequent work-up 50% of the time.

Although glucose testing does not always represent intentional screening, the patterns of testing were not random. Selected risk factors for diabetes (age 45 years and older, non-white ethnicity and family history of diabetes) independently increased the odds of glucose testing. Hypertension and hyperlipidemia were not associated with glucose testing, even though chemistry panels that include glucose are often ordered to monitor these diseases. These findings suggest that intentional screening for diabetes is occurring, even if physicians are not documenting this reason for glucose testing.

Glucose testing most closely resembled the guidelines of the ADA, based on the high rate of testing for eligible patients (97%) and the strong association of ADA-specific selection factors (such as non-white ethnicity and family history) with glucose testing. However, "adherence" to the CDC and USPSTF guidelines were also high, indicating that many of the risk factors used to select patients for screening are correlated with each other (age with hypertension, for example).

Our study builds on the findings of previous research. Ealovega and colleagues found that 69% of primary care patients received glucose testing over a 3-year period in a managed care setting [8], which is consistent with our finding of 78%. Ealovega and colleagues also found that only 38% of abnormal results received appropriate follow-up, consistent with our finding of 50% [8]. Other investigators found that glucose testing was associated with age [5] and family history of diabetes [6,7]. However, several of these previous analyses were limited by incomplete adjustment for potential confounders [5,7]. Ours was the only study to find an independent effect of ethnicity on the odds of glucose testing. Our finding that hypertension and hyperlipidemia were not independent predictors of glucose testing is in contrast to others' [8]. None of the previous studies specifically compared practice patterns to national screening guidelines.

Failure to recognize abnormal glucose values represents a missed opportunity to identify and treat incident diabetes or pre-diabetes, with timely treatment of pre-diabetes having the potential to prevent or delay the onset of diabetes [16]. That follow-up rates were higher for patients who had all visits with the same primary care physician is intriguing but based on a relatively small sample size and warrants replication in larger studies. There are several reasons why physicians may not act upon abnormal values: they may not have seen the values, may not have recognized their importance, or may have been giving their attention to competing demands. Indeed, a study that surveyed physicians about test result management in general found that only 52% of respondents reported keeping a record of tests that they had ordered and 83% acknowledged delays in recognizing and acting upon abnormal results, saying that they had reviewed at least 1 test result in the last 2 months that they "wished they had seen earlier" [17].

This study had several strengths. We used electronic orders for actual glucose tests, rather than patients' self-report of testing. We included an ethnically diverse patient population, with a proportion of ethnic minorities (41%) similar to that in the U.S. overall (37%) [18]. We followed patients over time, allowing for multiple primary care visits and approximating the 3-year screening interval suggested by the ADA. In addition, we used the rigorous statistical method of multiple imputation to account for missing data.

Several limitations merit discussion. First, height was missing for a substantial proportion of patients; however, results were similar when the multivariate model was performed with and without this variable. Second, we did not have enough power to adjust for clustering by physician, making it possible that some physicians' practice styles weighted the results more heavily than others'. Third, the study took place in one institution that may not be generalizable to other settings. Fourth, this study may actually underestimate the proportion of abnormal glucose values, because glucose values may be falsely low if the samples are not processed promptly. Fifth, patients who came for initial visits in 1999 may vary from patients who came only for subsequent visits that year. Sixth, it is possible that some patients with known diabetes did not report their condition at the initial visit, although this is unlikely because only 1 patient was diagnosed with diabetes – based on an abnormal glucose – after the initial visit; most patients diagnosed with diabetes were diagnosed after subsequent visits. Finally, as in all studies that examine medical records, some results were dependent on what physicians documented, although we minimized this by including in the analysis any glucose test ordered rather than only glucose tests for which screening was documented as the purpose.

## Conclusion

In conclusion, the majority of patients received glucose tests, and testing appeared to be informed by diabetes risk factors. These findings decrease the probability that undiagnosed diabetes is due to insufficient screening in primary care. However, lack of recognition and follow-up for abnormal glucose values may cause unnecessary delays in diagnosis and treatment. Interventions are needed not to increase the rate of testing but to trigger further evaluation of patients who have abnormal results.

## Competing interests

The authors declare that they have no competing interests.  The funding source was not involved in any aspect of  the study.

## Authors' contributions

LMK conceived and designed the study, contributed to data collection, had full access to all data collected, conducted data analysis, took responsibility for the integrity of the data and the accuracy of the data analysis, interpreted the data, and drafted and critically revised the manuscript. MAC contributed to study design, interpretation of the data, and critical revisions of the manuscript. DJB contributed to study design, interpretation of the data, and critical revisions of the manuscript. MV contributed to data collection, interpretation of the data, and critical revisions of the manuscript. AIM contributed to study design, interpretation of the data, and critical revisions of the manuscript. All authors read and approved the final manuscript.

## Pre-publication history

The pre-publication history for this paper can be accessed here:



## Figures and Tables

**Table 1:** Characteristics of patients without known diabetes

Characteristic	N = 301*
Age, mean years (sd)	40 (14)
Female, %	60
Ethnicity	
Non-Hispanic white, %	59
Black, %	19
Hispanic, %	11
Asian, %	8
Other, %	3
Insurance	
Commercial, %	87
Medicare, %	6
Medicaid, %	6
Self-pay, %	2
Family history of diabetes, %	22
History of gestational diabetes (women only), %	1
History of hypertension, %	10
History of high cholesterol, %	8
Height, mean inches (sd)	67 (4)
Weight, mean pounds (sd)	162 (41)

* Variables with missing data (and number of missing values): age (0), gender (0), ethnicity (50), insurance (7), family history (20), gestational diabetes (24 women), hypertension (9), high cholesterol (9), height (179), weight (24). Percentages may not total 100 due to rounding.

**Table 2:** Predictors of having at least 1 glucose test ordered, among 301 patients without known diabetes

	Odds Ratio (95% Confidence Interval)		
	
Predictor	Bivariate Models	Multivariate Model 1	Multivariate Model 2
Age (≥45 vs. <45 years)	14.00 (4.27 – 45.89)*	12.60 (3.51 – 45.23)*	12.36 (3.41 – 44.79)*
Gender (female vs. male)	0.54 (0.30 – 0.97)*	0.48 (0.22 – 1.04)†	0.45 (0.21 – 0.93)*
Ethnicity (non-white vs. white)	3.05 (1.55 – 6.01)*	3.66 (1.66 – 8.06)*	3.71 (1.68 – 8.20)*
Insurance (commercial vs. other)	0.41 (0.14 – 1.19)	1.61 (0.41 – 6.31)	1.76 (0.43 – 7.16)
Family history of diabetes (yes vs. no)	3.01 (1.27 – 7.11)*	3.14 (1.19 – 8.28)*	2.98 (1.12 – 7.93)*
Hypertension (yes vs. no)	7.92 (1.20 – 52.14)*	1.45 (0.18 – 11.60)	1.22 (0.15 – 9.82)
High cholesterol (yes vs. no)	1.30 (0.43 – 3.88)	0.72 (0.18 – 2.80)	0.71 (0.18 – 2.78)
Weight (per 10 pounds)	1.13 (1.04 – 1.22)*	1.07 (0.97 – 1.19)	---
Body mass index (per unit, kg/m^2^)	1.13 (1.06 – 1.21)*	---	1.08 (1.00 – 1.18)†
Number of visits observed (per visit)	1.32 (1.15 – 1.53)*	1.34 (1.13 – 1.60)*	1.33 (1.12 – 1.58)*
All visits with same physician (yes vs. no)	1.47 (0.85 – 2.55)	1.38 (0.69 – 2.75)	1.40 (0.70 – 2.80)

* p < 0.05. † p < 0.10.
